# Estimating the minimal clinically important difference of functional outcomes in spinal and bulbar muscular atrophy

**DOI:** 10.1007/s00415-026-14032-4

**Published:** 2026-07-31

**Authors:** Matteo Zanovello, Daniele Sabbatini, Federica Paredi, Alberto Romito, Sara Andreetta, Lorenzo Blasi, Giulia Musso, Angelo Poletti, Rosario Vasta, Manuela Basso, Raffaele Dubbioso, Maria Pennuto, Giacomo Maria Minicuci, Elena Pegoraro, Luca Bello, Gianni Sorarù

**Affiliations:** 1 Present Address: Neuromuscular Unit, Department of Neurosciences, University of Padova, 35128 Padova, Italy; 2 Present Address: Unit of Biostatistics, Epidemiology and Public Health, Department of Cardiac, Thoracic, Vascular Sciences, and Public Health, University of Padova, Padova, Italy; 3 Department of Medicine, University of Padova, Padova, Italy; 4 Dipartimento Di Scienze Farmacologiche E Biomolecolari “Rodolfo Paoletti”, Università Degli Studi Di Milano, Milano, Italy; 5 Department of Neuroscience “Rita Levi Montalcini”, University of Turin, Turin, Italy; 6 Centre for Integrative Biology, University of Trento, Trento, Italy; 7 Department of Neurosciences, Reproductive Sciences and Odontostomatology, University Federico II of Naples, Naples, Italy; 8 Department of Biomedical Sciences, University of Padova, Padova, Italy

**Keywords:** Neuromuscular diseases, Spinal and bulbar muscular atrophy, Functional rating scale, Minimal clinically important difference, Global rating of change, 6-min walk test

## Abstract

**Background and objectives:**

Spinal and bulbar muscular atrophy (*SBMA*) is a slowly progressive X-linked neuromuscular disorder for which disease-modifying therapies are under investigation. SBMA Functional Rating Scale (*SBMAFRS*), its subscale (mSBMAFRS), and Six-Minute Walk Test (*6MWT*) are commonly used trial endpoints, but thresholds for clinically meaningful change remain undefined. Minimal clinically important difference (*MCID*) estimates are needed to interpret longitudinal outcomes and inform trial design.

**Methods:**

We retrospectively analysed ambulatory, genetically confirmed SBMA patients. Eighty consecutive visit pairs from 44 patients included concurrent Global Rating of Change (*GRC*) assessments, and 47 visit pairs from 30 patients included concurrent 6MWT data. At follow-up, patients rated overall change since the previous visit on a 3‑level GRC (unchanged, slightly worse, much worse). Anchor-based MCIDs for worsening were derived from differences in change scores between GRC categories and compared with distribution-based estimates (0.5 baseline standard deviation). Sensitivity analyses and Monte Carlo resampling assessed robustness.

**Results:**

Anchor‑based MCID estimates for worsening were −1.13 and −1.46 points for the SBMAFRS total score, −0.53 and −1.09 points for the mSBMAFRS, and −34.5 and −32.4 m for the 6MWT. The mSBMAFRS showed the most consistent gradient across GRC categories and remained significant in sensitivity analyses. Distribution‑based MCIDs (2.30, 1.42 points and 61.85 m, respectively) were consistently larger than anchor‑based values. Age, disease duration, and CAG repeat length did not predict perceived worsening.

**Discussion:**

These data provide the first patient‑anchored MCID estimates for SBMA outcome measures, support use of the mSBMAFRS, and offer thresholds for responder definitions and sample-size calculations in future SBMA trials.

## Introduction

Spinal and bulbar muscular atrophy (*SBMA*), also known as Kennedy’s disease, is an X-linked neuromuscular disorder caused by a CAG repeat expansion exceeding 38 repeats in exon 1 of the androgen receptor (*AR*) gene encoding for a polyQ tract in the AR N-terminus [1]. The disease is characterized by slowly progressive lower motor neuron (*LMN*) and myofiber degeneration [2, 3]. The clinical picture manifests in adult males with slowly progressive limb and bulbar weakness, muscle atrophy and endocrine features, leading over decades to increasing disability and reduced quality of life.[4, 5].

Despite growing interest in disease‑modifying strategies (NCT05517603 NCT06411912 NCT06169046), clinical management and trial design in SBMA still rely largely on surrogate biomarkers such as disease‑specific functional rating scales and quantitative performance tests. Indeed, definitive clinical endpoints like loss of ambulation or need for ventilatory support occur late and are unsuitable as primary outcomes. Among these surrogate measures, the Spinal and Bulbar Muscular Atrophy Functional Rating Scale (*SBMAFRS*) [6] and its modified version (m‑SBMAFRS) [7] together with timed or distance‑based tests such as the 6‑Minute Walk Test (*6MWT*) [8] can detect subtle deterioration over relatively short intervals in SBMA [7]. However, these measures remain quantitative indices on arbitrary scales, and their interpretation in both trials and routine follow‑up critically depends on understanding how much change is actually meaningful for patients in their daily life. Importantly, regulatory guidance and payer evaluations are increasingly focused on whether trial outcomes reflect benefits that patients can feel and functionally appreciate [9].

Against this background, there is a clear need to derive patient‑reported estimates of clinically meaningful change for SBMA-specific biomarkers. In neuromuscular disorders, the concept of a minimal clinically important difference (*MCID*) has emerged as a key link between numerical change on a biomarker and a change that is relevant from the patient’s perspective [10]. Studies in Duchenne muscular dystrophy [11] and amyotrophic lateral sclerosis (*ALS*) [12] have shown that specific thresholds of change on functional scales or on the 6MWT correlate with patient- or clinician-reported worsening. Importantly, statistically significant group-level differences may fall below patients’ perceptible change, while even modest numerical effects can be clinically meaningful if they delay loss of a critical function.

For SBMA, the patient perspective has received comparatively little attention, and no MCID threshold exists for either the SBMAFRS or the 6MWT. To address this gap, we applied an anchor-based approach to derive MCID estimates for both instruments in a cohort of SBMA patients.

## Methods

### Cohort

We retrospectively enrolled genetically confirmed SBMA patients attending the Motor Neuron Disease Clinic of the University of Padova between January 2022 and March 2026. Inclusion required ambulatory status and completion of at least two scheduled follow-up visits to allow longitudinal assessment of functional variables. The interval between consecutive visits was recorded for each patient. The following patient data were collected: age and disease duration at first visit since muscle weakness onset, and CAG repeat number. Functional status was assessed using the SBMAFRS [6, 13] a disease-specific scale comprising five subscales: bulbar (5 items: speech, control of salivation, swallowing, tongue, puffing cheeks); upper-limb (2 items: writing, eating action); trunk (4 items: dressing activity, arising from a sitting position, arising from a supine position, bowing); lower-limb (2 items: walking, stairs); and respiratory (1 item: breathing). Each item is scored on a 5-point scale ranging from 0 (worst) to 4 (normal), yielding a maximum total score of 56. In addition to the SBMAFRS Total score, individual subscale scores were analysed separately. We also examined the mSBMAFRS, derived by including only lower limb and trunk subscales having lower variability and larger effect size compared with the others [7]. Motor performance was further assessed using the 6MWT, and assistive devices for walking were permitted. Anchor-based MCID estimates were based on within-patient change using the same device at consecutive visits, limiting any impact of device use.

To enable anchor-based MCID estimation, at each scheduled visit patients rated their perceived overall functional change since the previous visit using a 3-point Global Rating of Change (*GRC*) scale: 1 = unchanged (*U*), 2 = slightly worse (*SW*), 3 = much worse (*MW*). The GRC question was phrased as: ‘Compared with your last clinic visit, how would you rate your overall functional status today?’.

The study was approved by the Territorial Ethics Committee of the Central-Eastern Veneto Area (*CET*-*ACEV*), Italy (approval number: AOP 3874) and have therefore been performed in accordance with the ethical standards laid down in the 1964 Declaration of Helsinki and its later amendments. Written informed consent was obtained from every patient prior to study inclusion.

### Statistical analysis

Continuous variables are summarised as mean ± SD or median (IQR), categorical variables as *n* (%). All tests were two-sided with *α* = 0.05. Given the exploratory nature of the analyses, p-values were not adjusted for multiple comparisons. Between-visit changes (Δ) in each clinical measure were computed for all consecutive visit pairs within each patient, and the GRC rating recorded at the second visit of each pair was used as the external anchor. The anchor-based MCID was estimated as the difference in Δ between patients rating themselves as “slightly worse” and those rating themselves as “unchanged” (MCID-SW), and between those rating themselves as “much worse” and “unchanged” (MCID-MW). Because summed rating scales are ordinal, the mean between-group difference in Δ was used as the primary MCID estimate, with the corresponding median difference reported alongside. Differences in Δ across the three GRC categories were tested by Kruskal–Wallis. The distribution-based MCID was estimated as 0.5 × SD of the baseline value [14] Because individual patients could contribute multiple Δ observations to the primary analysis, the robustness of anchor-based estimates was first assessed by repeating the Kruskal–Wallis test on a single randomly selected visit pair per patient. To assess the robustness of the anchor-based MCID estimates under strict independence assumptions, we extended the single-pair sensitivity analysis using a Monte Carlo resampling approach: for each of 1000 iterations, one visit pair per patient was randomly selected without replacement, and the median Δ in each GRC group, together with the MW–U and SW–U median differences, was recomputed. The 2.5th and 97.5th percentiles of the resulting empirical distribution of median differences are reported as 95% percentile intervals. As a complementary sensitivity analysis that retains all visit pairs while accounting for within-patient correlation, the between-GRC differences in Δ were additionally modelled using generalised estimating equations (*GEE*; identity link, exchangeable working correlation, patient as clustering unit) and linear mixed-effects models (*LMM*) with a random patient intercept; the intraclass correlation coefficient (*ICC*) of the change scores was also estimated for each measure.

To assess whether baseline patient characteristics—CAG repeat length, age, and disease duration—predicted the probability of reporting clinical worsening (*GRC* = Slightly Worse or Much Worse) versus stability (*GRC* = Unchanged), we fitted univariable Generalised Estimating Equation (*GEE*) logistic regression models with an exchangeable working correlation structure and patient identifier as the clustering variable, to account for the inclusion of multiple visit pairs from the same patient. Odds ratios are reported per unit increase of each predictor, with 95% confidence intervals. All analyses were performed in R v4.4.

## Results

A total of 47 SBMA patients completed at least two scheduled visits, contributing to 80 concurrent GRC rating (34 “U”, 31 “SW”, 15 “MW”). The median duration of follow-up from first to last visit was 95.8 months (range 0.0–144.1), with a median interval between consecutive visits of 12.9 months (IQR 11.0–16.8), consistent with an annual assessment schedule. A total of 27 of the 47 patients used a walking aid at some point during follow-up. Patients’ characteristics at the first visit are summarized in Table 1.

**Table 1:** Baseline patients’ characteristics (*N* = 47)

Variable	*N*	Mean ± SD	Median	Range
Age at baseline (years)	47	50.8 ± 8.5	50.6	34.2–70.2
Disease duration (years)	47	7.7 ± 5.2	7.3	0.1–19.4
CAG repeat length	47	46.1 ± 2.8	46.0	41–51
SBMAFRS total	47	49.9 ± 4.6	51.0	38–56
SBMAFRS bulbar	47	17.3 ± 2.1	18.0	12–20
SBMAFRS upper limbs	47	7.5 ± 0.8	8.0	4–8
SBMAFRS trunk	47	14.9 ± 1.8	16.0	10–16
SBMAFRS lower limbs	47	6.3 ± 1.6	6.0	3–8
SBMAFRS respiratory	47	3.9 ± 0.3	4.0	3–4
mSBMAFRS (trunk + lower limbs)	47	21.2 ± 2.8	22.0	15–24
6MWT total distance (m)	44	421.8 ± 123.7	434.5	150–652

### Anchor-based MCID

**Fig. 1:**
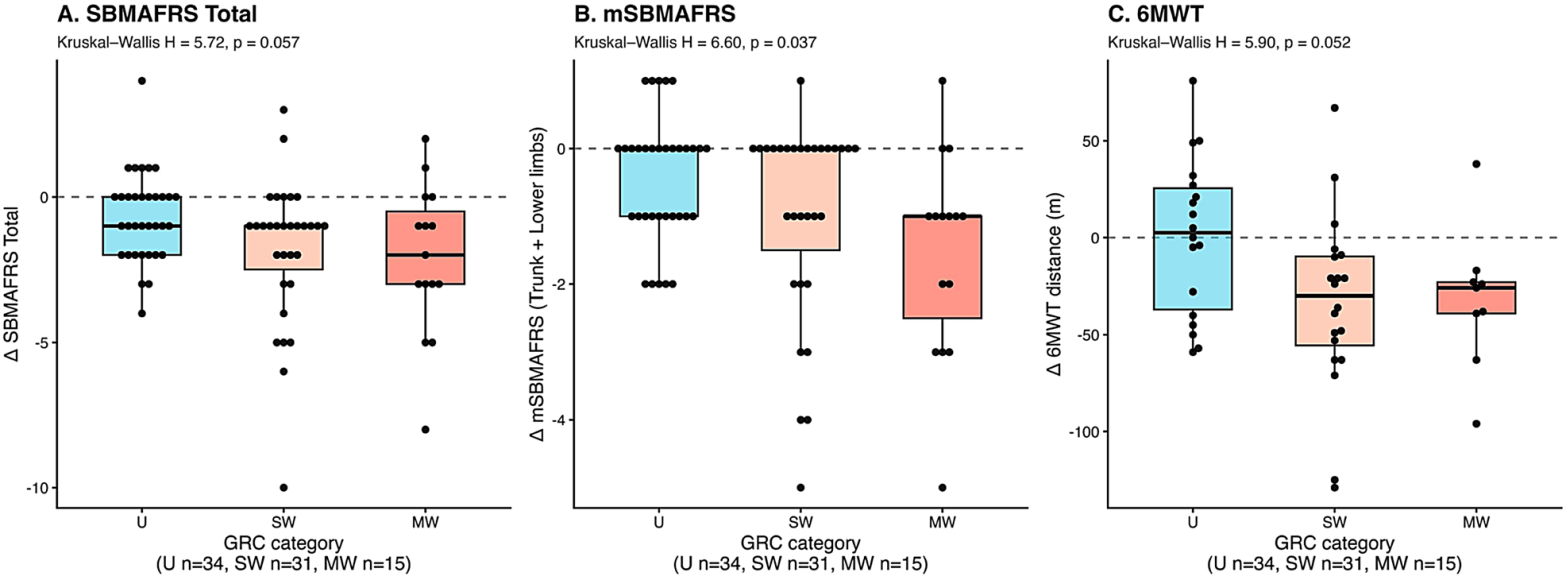
Distribution of change scores (Δ) across Global Rating of Change (*GRC*) categories for the primary outcome measures. Boxplots display median, interquartile range, and individual data points (beeswarm) for visit pairs rated as Unchanged (*U*, *n* = 34), Slightly Worse (*SW*, *n* = 31), or Much Worse (*MW*, *n* = 15). Negative values indicate clinical worsening. **A** SBMAFRS Total score; **B** mSBMAFRS composite (Trunk + Lower Limbs subscales); **C** Six-Minute Walk Test distance (*6MWT*, metres). A progressive decline in Δ is observed across GRC categories for all three measures, consistent with a dose–response relationship between perceived deterioration and measured functional change. Between-group differences reached statistical significance for the mSBMAFRS (Kruskal–Wallis *H* = 6.60, *p* = 0.037), with borderline results for SBMAFRS Total (*H* = 5.72, *p* = 0.057) and 6MWT (*H* = 5.90, *p* = 0.052)

To estimate anchor-based MCIDs, we analyzed change scores (Δ) across visit pairs within the three GRC categories (Fig. 1). Negative Δ values indicate worsening (i.e., lower SBMAFRS scores or shorter 6MWT distance at the follow-up visit compared with the preceding visit). For the SBMAFRS Total score, patients who rated themselves as *U* showed Δ values close to zero (mean −0.68, median −1.0), whereas SW and MW groups exhibited progressively larger declines (SW: mean −1.81, median −1.0; MW: mean −2.13, median −2.0), indicating a dose–response pattern consistent with increasing perceived deterioration (Fig. 1a). A similar monotonic gradient was observed for the mSBMAFRS (Fig. 1b) and, to a lesser extent, for the 6MWT distance (Fig. 1c). Between-group differences in Δ distributions were formally tested with the Kruskal–Wallis rank-sum test (Table 2). Statistically significant differences were detected for the mSBMAFRS (*H* = 6.60, *p* = 0.037), with borderline significance for the SBMAFRS total score (*H* = 5.72, *p* = 0.057) and the 6MWT (*H* = 5.90, *p* = 0.052). The ordinal trend was further confirmed by Spearman rank correlations between the three-level GRC anchor and the corresponding Δ, which were strongest for the SBMAFRS Total score (*ρ* = −0.26, *p* = 0.018), the mSBMAFRS (*ρ* = −0.27, *p* = 0.014), and the 6MWT (*ρ* = −0.33, *p* = 0.026). In contrast, the upper-limb (*UL*) and respiratory SBMAFRS subscales showed no significant between-group differences and near-zero Δ across all GRC categories.

**Table 2:** Anchor-based and distribution-based MCID estimates

Measure	*N* pairs	*N* patients	MCID-SW (mean, median)	MCID-MW (mean, median)	K-W H	K-W p	0.5 × SD	Monte Carlo 95%% PI (MCID-MW)	Monte Carlo 95%% PI (MCID-SW)
SBMAFRS Total	80	44	−1.13 (0.0)	−1.46 (− 1.0)	5.72	0.057	2.30	−2.5 to 0.0	−1.5 to 0.0
mSBMAFRS	80	44	−0.53 (0.0)	−1.09 (− 1.0)	6.60	0.037	1.42	−2.0 to 0.0	−1.0 to 1.0
6MWT (m)	47	30	−34.54 (− 32.5)	−32.39 (− 28.5)	5.90	0.052	61.85	−50.0 to 4.5	−57.5 to 1.0
SBMAFRS bulbar	80	44	−0.55 (0.0)	−0.50 (0.0)	4.78	0.092	1.03	−0.5 to 0.0	−1.0 to 0.0
SBMAFRS upper-limb	80	44	−0.14 (0.0)	0.15 (0.0)	1.75	0.417	0.39	−0.5 to 0.0	0.0 to 0.0
SBMAFRS trunk	80	44	−0.34 (0.0)	−0.79 (− 1.0)	5.54	0.063	0.88	−1.0 to 0.0	0.0 to 0.0
SBMAFRS lower-limb	80	44	−0.19 (0.0)	−0.31 (− 1.0)	1.86	0.395	0.78	−1.0 to 0.0	−1.0 to 0.0
SBMAFRS respiratory	80	44	0.12 (0.0)	0.02 (0.0)	2.04	0.360	0.17	0.0 to 0.0	0.0 to 0.0

The anchor-based MCID was operationally defined as the difference between the mean Δ in the SW group and the mean Δ in the U group (MCID-SW), with an analogous calculation for the MW-versus-*U* comparison (MCID-MW). For the SBMAFRS Total score, the MCID-SW was −1.13 points (median 0.0) and the MCID-MW was −1.46 points (median −1.0). For the mSBMAFRS, the corresponding estimates were −0.53 points (median 0.0) and −1.09 points (median −1.0), respectively. For the 6MWT, the two anchor-based estimates converged on approximately 33 m of clinically meaningful worsening(MCID-SW: mean −34.5 m, median −32.5 m; MCID-MW: mean −32.4 m, median −28.5 m), consistent across comparisons despite the smaller sample size available for this measure(*n* = 47 pairs with concurrent GRC and 6MWT data). A summary of all anchor-based and distribution-based MCID estimates is presented in Table 2.

### Distribution-based MCID

The distribution-based estimate (0.5 × SD of the baseline value)was 2.30 points for the SBMAFRS Total score, 1.42 points for the mSBMAFRS and 61.85 m for the 6MWT. Across all outcome measures, distribution-based MCID estimates systematically exceeded the corresponding anchor-based values by approximately 1.3- to 2-fold (Table 2).

### Sensitivity and monte carlo analyses

When Kruskal–Wallis tests were repeated using a single randomly selected visit pair per patient (*n* = 44 pairs), the mSBMAFRS remained statistically significant (*H* = 6.40, *p* = 0.041), while the SBMAFRS Total and 6MWT lost formal significance (*p* = 0.383 and *p* = 0.465, respectively), in keeping with reduced statistical power. A Monte Carlo resampling procedure (1000 iterations, one randomly selected visit pair per patient) confirmed the robustness of the mSBMAFRS anchor-based MCID-MW estimate, whose 95% percentile interval bordered zero at its upper bound (− 2.0 to 0.0 points). The 95% percentile intervals for the SBMAFRS Total (− 2.5 to 0.0) and 6MWT (− 50 to + 4.5 m) MCID-MW estimates included zero, indicating that the point estimates are directionally robust but statistically uncertain under strict independence assumptions.

To retain all visit pairs while accounting for repeated observations, the between-GRC differences in Δ were additionally modelled with GEE and linear mixed-effects models (*LMM*). The intraclass correlation of the change scores was negligible for the SBMAFRS-based measures (SBMAFRS Total ICC = 0.01; mSBMAFRS ICC ≈ 0) and moderate for the 6MWT (*ICC* = 0.31). The anchor-based point estimates were essentially unchanged: the much-worse-versus-unchanged contrast remained statistically supported for the mSBMAFRS (GEE −1.08, 95% CI −1.82 to −0.34, *p* = 0.004) and the SBMAFRS Total (GEE −1.45, 95% CI −2.72 to −0.18, *p* = 0.025), with concordant LMM estimates, whereas the 6MWT contrast remained non-significant (GEE −32.0 m, 95% CI −66.8 to + 2.9, *p* = 0.072). For the 6MWT, the only measure with non-negligible within-patient correlation, the slightly-worse-versus-unchanged estimate was attenuated under the correlation-aware models (− 20.5 m vs −34.5 m), whereas its non-significance was unchanged.

### Predictors of patient-perceived worsening

To assess whether patients’ characteristics, including CAG repeat length, age and disease duration at baseline, could predict the likelihood of a patient reporting clinical worsening at subsequent visits, we fitted univariable GEE logistic regression models with an exchangeable correlation structure to account for repeated visit pairs within the same patient. The outcome was binary: perceived worsening (*GRC* = Slightly Worse or Much Worse) versus perceived stability (*GRC* = Unchanged). Among 80 evaluable visit pairs from 44 patients, 46 (57.5%) were rated as worsened and 34 (42.5%) as unchanged. None of the three predictors was significantly associated with the probability of perceiving worsening: CAG repeat length (*OR* = 0.94 per repeat, 95% CI 0.77–1.17, *p* = 0.594),age at baseline (*OR* = 0.95 per year, 95% CI 0.89–1.02, *p* = 0.162), and disease duration (*OR* = 0.97 per year, 95% CI 0.87–1.09, *p* = 0.641).

## Discussion

The present study provides the first anchor-based MCID estimates for the SBMAFRS and the 6MWT in SBMA, establishing a clinically meaningful link between changes in these functional measures and changes that are relevant from the patient's perspective. Our findings suggest that a decline of approximately 1–2 points on the SBMAFRS Total score, approximately 1 point on the mSBMAFRS, and approximately 33 m on the 6MWT represent thresholds of meaningful worsening as perceived by patients, offering a concrete reference for the interpretation of functional outcomes in future natural history studies and clinical trials.

The SBMAFRS Total score showed an anchor-consistent gradient in the pooled analysis, supporting construct validity, in line with previous psychometric validation work by Hashizume et al. [6]. However, this finding was attenuated in the resampling-based sensitivity analyses (single-pair and Monte-Carlo), which retain only one observation per patient and are therefore underpowered, although it was supported by the correlation-aware models retaining all observations (GEE and linear mixed-effects; see Results). Because the within-patient correlation of the change scores was negligible for the SBMAFRS-based measures, the assumption of independence had little practical impact on these outcomes; the two approaches are complementary, the pooled anchor-based analysis quantifying the clinically interpretable magnitude of the MCID and the correlation-aware models confirming that this signal persists once within-patient dependence is taken into account. In contrast, the mSBMAFRS demonstrated a more reliable behaviour, with statistically significant between-group differences and a consistent gradient across GRC categories, such that patients who perceived greater worsening showed correspondingly larger declines on the scale. This robustness was further confirmed by sensitivity analyses preserving independence of observations, in which the mSBMAFRS remained the only measure to retain statistical significance. This finding supports the preferential use of the mSBMAFRS over the SBMAFRS Total score as a more sensitive and clinically meaningful outcome measure in future natural history studies and interventional trials. Notably, Huggett et al. [7] had previously identified the mSBMAFRS as preferable to the SBMAFRS Total score on different grounds, namely its lower variability and greater consistency across international clinical sites; our anchor-based results extend this evidence by providing an independent, patient-centred rationale for the same conclusion. Of note, the individual trunk and lower-limb subscales did not reach significance when tested separately (*p* = 0.063 and *p* = 0.395, respectively), yet their combination did. This may indicate that each subscale captures complementary aspects of clinically relevant motor decline, so that their combination enhances sensitivity to change. This is consistent with the known pattern of muscle involvement in SBMA, where either proximal lower-limb and pelvic girdle muscles are preferentially affected early in the disease course, as demonstrated by whole-body MRI studies showing that fat infiltration initiates in the posterior calf and progresses to the posterior thigh and pelvic muscles before involving more distal or upper extremity compartments [15]. The remaining SBMAFRS subscales—bulbar, upper-limb, and respiratory—showed no significant difference between anchor groups and yielded anchor-based estimates that were inconsistent or near zero. Rather than representing a methodological failure, the absence of meaningful signal in these domains reflects their limited clinical relevance in ambulatory patients with SBMA: bulbar and respiratory involvement are hallmarks of advanced disease stages [16] and are unlikely to capture meaningful change within a cohort predominantly composed of patients at earlier functional levels. Corroborating this view, a large patient-reported study of SBMA identified lower-limb function and gait as the symptomatic domains with the greatest impact on daily life, whereas bulbar, upper-limb, and respiratory features ranked among the least impactful [4]. Therefore, dedicated studies to non-ambulatory or more advanced SBMA patients will be needed to establish appropriate MCID across the full spectrum of disease severity.

For the 6MWT, a preliminary anchor-based estimate of MCID suggests a threshold of approximately 33 m, with closely consistent values obtained across the two anchor comparisons (*SW* = −34.5 m and *MW* = −32.4 m). This convergence may partly reflect an inherent limitation of the 3-point GRC anchor, which captures the direction of perceived change but does not quantify its magnitude. As noted by Kamper et al. [17], scales with very few response categories have reduced discriminative ability and lower reliability, with a consequent risk of overlap between adjacent severity groups. Empirical evidence suggests that GRC scores are influenced by a patient's current health status more than by actual functional transition, which may further reduce separation between the SW and MW groups. Nonetheless, the consistency of the threshold across both comparisons supports its plausibility as a meaningful estimate. The between-group difference narrowly missed conventional statistical significance (*p* = 0.052), and the sensitivity analyses were constrained by the smaller subset of patients with available 6MWT data. The estimate of ~33 m should therefore be interpreted as preliminary; validation in larger, prospectively designed cohorts will be necessary before it can be applied with confidence in clinical or trial settings.

Distribution-based MCID estimates were consistently higher than anchor-based estimates across all three outcomes, by approximately one- to twofold. This discrepancy is not unexpected: it stems from a fundamental difference in what the two approaches are designed to measure. Anchor-based estimates identify the smallest change that patients themselves perceive as clinically meaningful, and are therefore considered the reference standard for patient-centred outcome interpretation. Distribution-based estimates, by contrast, quantify the change required to exceed inherent measurement variability, a heuristic approach formalized by Norman et al. [14] in a rare, slowly progressive disease such as SBMA, this variability is inevitably amplified by the clinical heterogeneity of the population. Importantly, as demonstrated by Franceschini et al. [18] different MCID calculation methods can yield widely divergent threshold values, with direct consequences for the interpretation of treatment success. We therefore adopted a triangulation approach, treating the anchor-based estimates as the primary clinically meaningful thresholds and the distribution-based estimates as complementary indicators of measurement noise.

Contextualising the anchor-based MCID estimates derived in this study against published natural history data reveals an important challenge for trial design. Hashizume et al. [6] reported a mean annual decline of approximately 1.5 points on the SBMAFRS Total score, while Huggett et al. [7] in a large international meta-analysis of the global SBMA dataset, documented a mean decline of approximately 0.9 points per year on the mSBMAFRS and approximately 24 m per year on the 6MWT, with the SBMAFRS showing only moderate responsiveness over a 1-year interval. Takeuchi et al. [8] further documented an annual 6MWT decline of approximately 36 m in their SBMA cohort. Notably, the expected annual rates of progression derived from natural history data are quite close to the corresponding anchor-based MCID thresholds identified in the present study, suggesting that patients undergoing natural disease course may change by approximately the minimum amount they themselves perceive as meaningful over a single year. This near-equivalence is particularly relevant given that assessments in our cohort were performed at annual intervals, leaving little statistical margin to detect a treatment effect within a standard 12-month follow-up. Consistently, the relatively high proportion of visit-pairs in which patients rated themselves as unchanged (42%) suggests that gradual annual decline approximating the MCID threshold may frequently fall below the level of subjective perception within a single follow-up interval. The well-documented heterogeneity of individual progression trajectories, further characterised in our own natural history cohort [19] compounds this challenge, as between-patient variability reduces the power to detect treatment effects based on mean group differences alone. Taken together, these observations highlight a fundamental tension in trial design for a slowly progressive rare disease such as SBMA: the threshold of change that is meaningful to the patient may not be statistically detectable within the timeframe of a conventional clinical trial unless sample sizes are large or follow-up is extended.

The absence of a significant association between perceived worsening and key baseline characteristics, namely CAG repeat length, age, and disease duration, suggests that the MCID estimates derived in this study are broadly applicable across ambulatory patients with SBMA, regardless of disease stage or genetic background. The ambulatory status required for inclusion in our cohort further mirrors the eligibility criteria commonly adopted in interventional trials in SBMA (NCT05517603 NCT06411912 NCT06169046), supporting the direct applicability of these estimates to a trial setting.

### Limitations

Several limitations of the present study warrant acknowledgement. First, anchor-based MCID methods are inherently susceptible to recall bias, as global ratings of change may reflect patients’ current health status rather than an accurate retrospective appraisal of change since the previous visit [14] a limitation that may be even amplified in our study by the annual interval between visits. Second, the SBMAFRS scores are ordinal in nature [6] although treating summed rating scales as continuous variables is standard practice in neuromuscular research, the assumption of equal inter-point distances may not hold, and non-parametric MCID estimates (medians) were therefore reported alongside parametric estimates. Third, the relatively small sample size and retrospective design limited the standardization of clinical assessments across visits and reduced the precision of the anchor-based MCID estimates, as reflected by the wider Monte Carlo percentile intervals observed for the SBMAFRS Total score and the 6MWT. Moreover, this was a single-centre study; together with the limited sample size, this restricts the generalisability of our findings, and the proposed thresholds should be regarded as provisional estimates to be confirmed in larger, multicentre cohorts. The between-visit interval was approximately one year; given the slow progression of SBMA, the small functional change accrued over such an interval limits the precision and reliability of the anchor-based estimates. Finally, because the GRC used here captured only stability and worsening, with no patient reporting improvement during follow-up, the present MCID estimates pertain specifically to the detection of clinically meaningful deterioration; as disease-modifying and rehabilitative interventions capable of producing functional improvement emerge in SBMA, future studies should employ a bidirectional anchor to derive MCID estimates for improvement as well (e.g., 7- to 15-point) [17].

## Conclusions

We provide the first anchor-based MCID estimates for the SBMAFRS, mSBMAFRS and 6MWT in SBMA.

Given the modest sample size and single-centre design, these estimates should be considered preliminary; nonetheless, they offer empirically grounded starting points for responder definitions and sample size assumptions, which must be prospectively validated in multicentre SBMA trials before adoption in confirmatory settings. They should also be incorporated prospectively alongside emerging patient‑reported outcome measures, such as the recently validated SBMA Health Index [20], to move towards a more fully patient‑centred outcome framework for SBMA.

## Data Availability

The datasets generated and/or analysed during the current study are not publicly available due to privacy reasons but are available from the corresponding author on reasonable request.
